# Polyphenol intake and mortality risk: a re-analysis of the PREDIMED trial

**DOI:** 10.1186/1741-7015-12-77

**Published:** 2014-05-13

**Authors:** Anna Tresserra-Rimbau, Eric B Rimm, Alexander Medina-Remón, Miguel A Martínez-González, M Carmen López-Sabater, María I Covas, Dolores Corella, Jordi Salas-Salvadó, Enrique Gómez-Gracia, José Lapetra, Fernando Arós, Miquel Fiol, Emili Ros, Lluis Serra-Majem, Xavier Pintó, Miguel A Muñoz, Alfredo Gea, Valentina Ruiz-Gutiérrez, Ramón Estruch, Rosa M Lamuela-Raventós

**Affiliations:** 1 Nutrition and Food Science Department, XaRTA, INSA, Pharmacy School, University of Barcelona, Barcelona, Spain; 2 CIBER CB06/03 Fisiopatología de la Obesidad y la Nutrición (CIBERObn), Institute of Health “Carlos III”, Government of Spain, Madrid, Spain; 3 Harvard Medical School and Harvard School of Public Health, Boston, MA, USA; 4 Department of Preventive Medicine and Public Health, School of Medicine, University of Navarra, Pamplona, Spain; 5 Cardiovascular Epidemiology Unit, Municipal Institute for Medical Research (IMIM), Barcelona, Spain; 6 Department of Epidemiology, Preventive Medicine and Public Health, School of Medicine, University of Valencia, Valencia, Spain; 7 Human Nutrition Unit, School of Medicine, IISPV, University Rovira i Virgili, Reus, Spain; 8 Department of Epidemiology, School of Medicine, University of Malaga, Málaga, Spain; 9 Department of Family Medicine, Primary Care Division of Sevilla, San Pablo Health Center, Sevilla, Spain; 10 Department of Cardiology, Hospital Txangorritxu, Vitoria, Spain; 11 Institut Universitari d´Investigació en Ciències de la Salut (IUNICS), Palma de Mallorca, Spain; 12 Lipid Clinic, Endocrinology and Nutrition Service, Institut d’Investigacions Biomédiques August Pi i Sunyer (IDIBAPS), Hospital Clinic, Barcelona, Spain; 13 Department of Clinical Sciences, University of Las Palmas de Gran Canaria, Palmas de Gran Canaria, Spain; 14 Lipid Unit, Department of Internal Medicine, IDIBELL-Hospital Universitari de Bellvitge, L'Hospitalet de Llobregat, FIPEC, Barcelona, Spain; 15 Primary Care Division Catalan Institute of Health, Barcelona, Spain; 16 Nutrition and Lipids Metabolism, Instituto de la Grasa, Consejo Superior de Investigaciones Cientificas, Sevilla, Spain; 17 Department of Internal Medicine, Hospital Clínic, IDIBAPS, University of Barcelona, Barcelona, Spain

**Keywords:** Polyphenol intake, All-cause mortality, PREDIMED, Mediterranean diet, Stilbenes, Lignans

## Abstract

**Background:**

Polyphenols may lower the risk of cardiovascular disease (CVD) and other chronic diseases due to their antioxidant and anti-inflammatory properties, as well as their beneficial effects on blood pressure, lipids and insulin resistance. However, no previous epidemiological studies have evaluated the relationship between the intake of total polyphenols intake and polyphenol subclasses with overall mortality. Our aim was to evaluate whether polyphenol intake is associated with all-cause mortality in subjects at high cardiovascular risk.

**Methods:**

We used data from the PREDIMED study, a 7,447-participant, parallel-group, randomized, multicenter, controlled five-year feeding trial aimed at assessing the effects of the Mediterranean Diet in primary prevention of cardiovascular disease. Polyphenol intake was calculated by matching food consumption data from repeated food frequency questionnaires (FFQ) with the Phenol-Explorer database on the polyphenol content of each reported food. Hazard ratios (HR) and 95% confidence intervals (CI) between polyphenol intake and mortality were estimated using time-dependent Cox proportional hazard models.

**Results:**

Over an average of 4.8 years of follow-up, we observed 327 deaths. After multivariate adjustment, we found a 37% relative reduction in all-cause mortality comparing the highest versus the lowest quintiles of total polyphenol intake (hazard ratio (HR) = 0.63; 95% CI 0.41 to 0.97; *P* for trend = 0.12). Among the polyphenol subclasses, stilbenes and lignans were significantly associated with reduced all-cause mortality (HR =0.48; 95% CI 0.25 to 0.91; *P* for trend = 0.04 and HR = 0.60; 95% CI 0.37 to 0.97; *P* for trend = 0.03, respectively), with no significant associations apparent in the rest (flavonoids or phenolic acids).

**Conclusions:**

Among high-risk subjects, those who reported a high polyphenol intake, especially of stilbenes and lignans, showed a reduced risk of overall mortality compared to those with lower intakes. These results may be useful to determine optimal polyphenol intake or specific food sources of polyphenols that may reduce the risk of all-cause mortality.

**Clinical trial registration:**

ISRCTN35739639.

## Background

Diet and lifestyle are crucial in the prevention of chronic illnesses and therefore substantially lower all-cause mortality in most westernized countries. There is evidence that the Mediterranean diet (MedDiet), a well characterized dietary pattern, is associated with longevity and improved quality of life by reducing the risk of the most frequent chronic diseases such as cardiovascular diseases (CVD), metabolic syndrome, age-related cognitive impairment, type 2 diabetes mellitus (T2DM), cancer and also all-cause mortality [1,2]. The MedDiet is rich in fruits and vegetables, olive oil, nuts, legumes, whole-wheat bread and fish, and wine is consumed in moderate amounts during meals [2]. With respect to nutrients, the MedDiet is very rich in mono- and polyunsaturated fatty acids [3] and also in polyphenols, which are bioactive compounds mainly found in plant foods and plant-derived beverages such as coffee, tea and red wine.

Several studies have examined the association between intake of certain polyphenol subgroups and their sources, and the incidence of chronic degenerative diseases [4], as well as their effects on blood pressure, lipid profile, and endothelial and platelet function [5-7]. If polyphenol intake does protect against the development of chronic diseases such as CVD, cancer or T2DM, we hypothesized that a greater consumption of polyphenols would contribute to lower the risk of all-cause mortality and provide a greater life expectancy.

To date, the association between specific groups of polyphenols and mortality has been described [8], but to our knowledge, neither total polyphenol intake nor that of the different polyphenol subgroups, have been associated with all-cause mortality. We therefore embarked on a study to evaluate the association between the intake of total polyphenols and polyphenol subgroups and the risk of overall mortality, using the Phenol-Explorer database [9] to estimate the polyphenol intake recorded by the food frequency questionnaires (FFQ) administered yearly in the PREDIMED (*Prevención con Dieta Mediterránea*) trial. These results may be useful to determine optimal polyphenol intake or specific food sources of polyphenols that may reduce the risk of all-cause mortality among subjects at high cardiovascular risk.

## Methods

### The PREDIMED study

The PREDIMED study was a parallel-group, randomized, multicenter, controlled feeding trial aimed at assessing the effects of the MedDiet in the primary prevention of cardiovascular disease. Details of the recruitment method and study design have been described elsewhere [10]. The eligible participants were 7,447 community-dwelling men (55 to 80 years) and women (60 to 80 years) from Spain, who had no cardiovascular disease at enrollment but were at high risk: they had either T2DM or at least three of the following major risk factors: smoking, hypertension, dyslipidemia, overweight or obesity, or a family history of premature coronary heart disease. Starting on 1 October 2003, the eligible participants were randomized in a 1:1:1 ratio to one of three dietary intervention groups: 1) MedDiet supplemented with extra-virgin olive oil (EVOO), 2) MedDiet supplemented with mixed nuts or 3) control diet (low-fat diet). The trial was stopped after a median follow-up of 4.8 years due to the benefit of the MedDiets with respect to major cardiovascular events: myocardial infarction, stroke or death from cardiovascular causes (analysis performed by the Drug and Safety Monitoring Board of the trial), compared to a control low-fat group [2]. All participants provided written informed consent, and the study protocol was approved by the Institutional Review Boards of the participating centers (Hospital Clínic of Barcelona (coordinating centre), Universities of Barcelona, Valencia, Rovira-Virgili, Málaga and Las Palmas, Municipal Institute for Medical Research, Primary Care Division of Barcelona and Sevilla, Institute of Research in Health Sciences (IUNICS) at Palma de Mallorca, Hospital Txangorritxu of Vitoria, and University Hospital of Bellvitge) and registered [11].

### Study population and characteristics

The present study was conducted as a re-analysis of an intervention feeding study using polyphenol intake as the exposure. Data came from all participants of the PREDIMED trial, but we excluded 247 individuals with an inadequate FFQ at baseline and 28 with a total energy intake out of the predefined limits (that is, daily energy intake <500 or >3,500 for women and <800 or >4,000 kcal/d for men; n = 28) [12]. Therefore, data from 7,172 participants were available for this analysis.

Participants filled out the following questionnaires at baseline and yearly thereafter: a validated 14-point score questionnaire on adherence to the traditional MedDiet [13], a validated 137-item FFQ [14], and a general questionnaire which included data on lifestyle habits, concurrent diseases and medication used.

### Polyphenol intake and dietary assessment

At baseline and yearly thereafter, trained dietitians completed the validated 137-item FFQ [14] in a face-to-face interview with the participant. Energy and nutrient intake were estimated from the FFQ by multiplying the frequency of consumption by the average portion size using Spanish food composition tables.

In a previous study conducted by our group, total polyphenol excreted in spot urine samples was validated as a biomarker of total polyphenol intake from FFQ in a clinical trial (r = 0.48, *P* <0.01) and in a cross-sectional study (r = 0.26, *P* = 0.04) [15]. The Phenol-Explorer database [9] was used to obtain information about polyphenol content in foods. This database included 516 polyphenols contained in 456 foods [16] at the time of our analysis, being the most complete database currently available for polyphenol content. Correspondence between food items in the FFQ and the Phenol-Explorer database has been described previously [17]. Individual polyphenol intake was calculated by multiplying the content of each polyphenol in a particular food item (mg/g) by the daily consumption of this food item (g/day) and then summing the product across all food items. Total polyphenol intake was the sum of all individual polyphenol intakes.

Polyphenol and other nutrient intakes were adjusted for total energy intake because it is associated with disease risk and is usually proportional to most nutrient intake [18]. To conduct the analyses, we also used weighted cumulative averages, that is, the polyphenol intake of a given year was the average between the intake of that year and the average of the previous years.

### Ascertainment of the outcome

Information on mortality was updated yearly by the end-point adjudication committee, whose members were unaware of dietary intakes or intervention assignments. The sources of information were the following: yearly questionnaires and examinations from all participants, family physicians, yearly review of medical records and linkage to the National Death Index. All outcomes were reported between 1 October 2003 and 1 December 2010.

### Statistical analyses

We calculated the weighted cumulative average of polyphenol intake at each yearly visit to represent long-term polyphenol intake. Polyphenols and other food and nutrient intake were adjusted for total calories using the residual method. Non-dietary covariates such as smoking, body mass index (BMI), physical activity and medication use were updated yearly.

The baseline characteristics of the 7,172 participants were distributed by quintiles of total polyphenol intake. Data were presented as means (±SD) for continuous variables and frequencies, and percentages for categorical variables. We used one-factor ANOVA or Pearson chi-squared tests to compare the quantitative or categorical baseline characteristics of the study participants across quintiles of baseline polyphenol intake. Person-time for each participant was calculated as the time between randomization and the date of death, the date when completing the last interview, 1 December 2010 or date at death, whichever came first. To assess the risk of total mortality by quintiles of polyphenol intake, we ran time-dependent Cox proportional hazard regressions with updated diet and covariates. The referent group was the lowest quintile of polyphenol intake. Results are expressed as hazard ratios (HRs) with 95% confidence intervals (CIs). To show the crude differences in death rates by groups of polyphenol intake, we performed a Nelson Aalen survival function, a non-parametric estimator of the survival function for censored data.

Moreover, we used likelihood ratio tests of interaction in stratified analyses to study the possible interactions among the main risk factors and, as sensitivity analyses, we estimated the fully adjusted HR, excluding participants with less than one or two years of follow-up.

### Covariates

To take into account the potential differences in risk factors, all Cox proportional hazard analyses were carried out with stratification for recruitment center, sex and intervention group. In model 2, we adjusted for sex, age (<60, 60 to 64.9, 65 to 69.9, 70 to 74.9, >/=75 years), smoking status (never, past and current: cigarettes (<5, 5 to 19, >20 per day) or cigars and pipes (<3, 3 to 6, >6 per day)), BMI (<25, 25 to 29.9, or >/=30 Kg/m^2^), baseline diabetes, alcohol consumption (0, 0.1 to 14.9, 15 to 29.9, >/=30 g/day), total energy intake (continuous variable), physical activity (continuous variable), family history of CVD and/or cancer, aspirin use, antihypertensive drug use, use of cardiovascular medication, use of oral hypoglycemic agents, insulin and other medication. In model 3, we additionally adjusted for intake of protein, saturated fatty acids, polyunsaturated fatty acids, monounsaturated fatty acids and cholesterol. We did not include in the model other variables that did not change the HR by 10% or more.

Statistical analyses were conducted using SAS software, version 9.3 (SAS Institute, Inc., Cary, NC, USA). All t tests were two-sided and *P*-values below 0.05 were considered significant.

## Results

The baseline characteristics of participants are shown by quintiles of energy-adjusted total polyphenol intake in Table 1. Participants with a greater intake of total polyphenols had a closer adherence to the traditional MedDiet. They also tended to be more physically active, consume more alcoholic beverages (mostly wine and beer) and to have less hypertension. On the contrary, the prevalence of hypercholesterolemia was higher in those who consumed more polyphenols at baseline and they were more likely to be smokers. The groups did not differ in terms of diabetes status, use of medication and distribution into the three arms of the trial.

**Table 1:** Baseline characteristics according to quintiles of total polyphenol intake at baseline (energy-adjusted)

	**Q1**	**Q2**	**Q3**	**Q4**	**Q5**	***P-*** value*
	**(n = 1,434)**	**(n = 1,435)**	**(n = 1,434)**	**(n = 1,435)**	**(n = 1,434)**	
Polyphenol intake, mean (cutoff values), mg/d	483 (<642)	674 (642 to 749)	794 (750 to 852)	937 (853 to 995)	1,235 (>995)	
Sex, women	836 (58.3)	924 (64.4)	712 (60.8)	803 (56.0)	648 (45.2)	<0.0001
Age, mean (SD), y	67.6 (6.2)	67.4 (6.1)	67.4 (5.9)	66.9 (6.0)	66.2 (6.1)	<0.0001
BMI, mean (SD), Kg/m^2^	30.0 (3.7)	30.3 (3.7)	29.7 (3.5)	29.7 (3.7)	29.6 (3.5)	<0.0001
Current smoker	217 (15.1)	210 (14.6)	194 (13.5)	265 (18.5)	317 (22.1)	<0.0001
Former smoker	273 (19.0)	263 (18.3)	317 (22.1)	319 (22.2)	413 (28.8)	
Sports/exercise, mean (SD), MET-h/d	3.37 (3.56)	3.62 (3.83	3.77 (3.66)	4.05 (4.25)	4.59 (4.54)	<0.0001
Diabetes	706 (49.2)	680 (47.4)	712 (49.6)	704 (49.1)	668 (46.6)	0.40
Hypertension	1,230 (85.8)	1,224 (85.3)	1,192 (83.1)	1,166 (81.3)	1,117 (77.9)	<0.0001
Hypercholesterolemia	983 (68.6)	1,018 (70.9)	1,053 (73.4)	1,065 (74.2)	1,069 (74.6)	0.001
Hypolipidemic drug use	660 (46.1)	670 (46.7)	712 (49.7)	716 (50.1)	706 (49.5)	0.09
Antihypertensive drug use	1,071 (74.7)	1,095 (76.4)	1,027 (71.7)	1,030 (72.0)	994 (69.7)	0.0004
Cardiovascular drugs use	118 (8.5)	114 (8.2)	120 (8.6)	110 (7.9)	109 (7.9)	0.94
Insulin use	90 (6.3)	87 (6.1)	115 (8.0)	95 (6.6)	99 (6.9)	0.26
Anti-diabetes drug use, other than insulin	463 (32.3)	454 (31.7)	478 (33.4)	465 (32.5)	439 (30.8)	0.65
Aspirin use	302 (21.1)	326 (22.8)	337 (23.5)	318 (22.2)	324 (22.7)	0.63
Int. Group: MedDiet-EVOO	489 (34.1)	506 (35.3)	477 (33.6)	473 (33.0)	517 (36.1)	0.001
Int. Group: MedDiet-nuts	444 (31.0)	467 (32.5)	454 (31.7)	491 (34.2)	519 (36.2)	
**Mean daily intake:**						
Total energy intake, mean (SD), Kcal/d	2,397 (642)	2,180 (589)	2,161 (540)	2,229 (563)	2,369 (577)	<0.0001
Carbohydrates, mean (SD), g/d	240 (45)	237 (39)	235 (37)	234 (41)	236 (45)	0.006
Protein, mean (SD), g/d	91.9 (15.1)	92.4 (13.8)	92.4 (13.2)	91.5 (13.6)	90.6 (14.9)	0.004
SFA, mean (SD), g/d	26.1 (6.7)	25.4 (5.7)	25.1 (5.3)	24.9 (5.5)	23.5 (5.8)	<0.0001
MUFA, mean (SD), g/d	49.0 (12.2)	48.8 (10.6)	48.8 (10.7)	48.7 (11.3)	46.6 (11.2)	<0.0001
PUFA, mean (SD), g/d	15.6 (5.8)	15.9 (5.1)	15.8 (5.0)	15.8 (5.2)	15.0 (5.2)	<0.0001
Fiber, mean (SD), g/d	21.5 (6.1)	23.9 (6.4)	25.5 (6.7)	26.6 (7.4)	29.4 (8.9)	<0.0001
Total cholesterol, mean (SD), mg/d	372 (121)	367 (103)	368 (107)	360 (94)	354 (122)	<0.0001
Alcohol, mean (SD), g/d	4.10 (10.9)	6.3 (10.1)	7.6 (10.5)	9.3 (12.8)	14.6 (18.9)	<0.0001
Vegetables, mean (SD), g/d	296 (140)	319 (127)	338 (139)	351 (142)	369 (169)	<0.0001
Fruits, mean (SD), g/d	240 (133)	319 (145)	364 (157)	404 (172)	521 (245)	<0.0001
Legumes, mean (SD), g/d	20.5 (15.3)	20.7 (15.2)	20.3 (10.9)	20.6 (12.4)	20.6 (13.0)	0.93
Dairy products, mean (SD), g/d	398 (226)	391 (216)	389 (208)	380 (219)	353 (217)	<0.0001
Cereals, mean (SD), g/d	247 (98)	233 (81)	227 (78)	219 (79)	209 (80)	<0.0001
Meat or meat products, mean (SD), g/d	135 (60)	132 (54)	132 (50)	130 (50)	129 (55)	0.03
Fish, mean (SD), g/d	94.3 (53.3)	99.9 (46.8)	101 (51.5)	99.6 (45.0)	102 (49.2)	0.0005
Sugar-sweetened soft drinks, mean (SD), g/d	25.0 (84.3)	19.7 (63.3)	17.8 (55.8)	15.4 (56.1)	12.6 (46.3)	<0.0001
Coffee, mean (SD), g/d	25.8 (36.3)	43.6 (40.1)	55.2 (42.9)	70.3 (49.2)	90.1 (63.8)	<0.0001
14-points MedDiet questionnaire score, mean (SD)	8.2 (1.9)	8.5 (1.9)	8.7 (1.9)	8.7 (1.9)	9.2 (1.8)	<0.0001
**Risk factors:**						
Waist-to-height ratio, mean (SD)	0.64 (0.06)	0.63 (0.07)	0.63 (0.06)	0.62 (0.06)	0.62 (0.06)	<0.0001
Systolic BP, mean (SD), mmHg	150 (19)	151 (19)	149 (19)	148 (18)	148 (18)	0.01
Diastolic BP, mean (SD), mmHg	83 (10)	84 (9.8)	82 (9.6)	82 (9.8)	83 (9.6)	0.003
Hearth rate, mean (SD), beats/min	71.7 (11.0)	71.2 (10.9)	70.7 (11.1)	70.0 (10.5)	70.5 (10.5)	0.02
Glucose (n = 4,311), mean (SD), mg/dL	118 (41)	116 (39)	122 (42)	123 (43)	123 (43)	0.0007
Cholesterol (n = 4,286), mean (SD), mg/dL	202 (36)	206 (38)	207 (39)	208 (38)	207 (36)	0.003
HDL (n = 4,236), mean (SD), mg/dL	50 (11)	51 (11)	51 (11)	52 (12)	52 (11)	0.007
Triglycerides (n = 4,291), mean (SD), mg/dL	130 (67)	133 (74)	137 (79)	130 (63)	138 (80)	0.06

BMI*,* Body Mass Index; BP, Blood pressure; MedDiet-EVOO, Mediterranean Diet supplemented with extra virgin olive oil; MedDiet-nuts*,* Mediterranean Diet supplemented with nuts; SFA, Saturated fatty acids; MUFA, Monounsaturated fatty acids; PUFA, Polyunsaturated fatty acids; HDL, High density lipoprotein.

Data are expressed as No. (%) unless otherwise indicated.

^*^P-values calculated by analysis of variance or χ^2^ tests.

**Figure 1:**
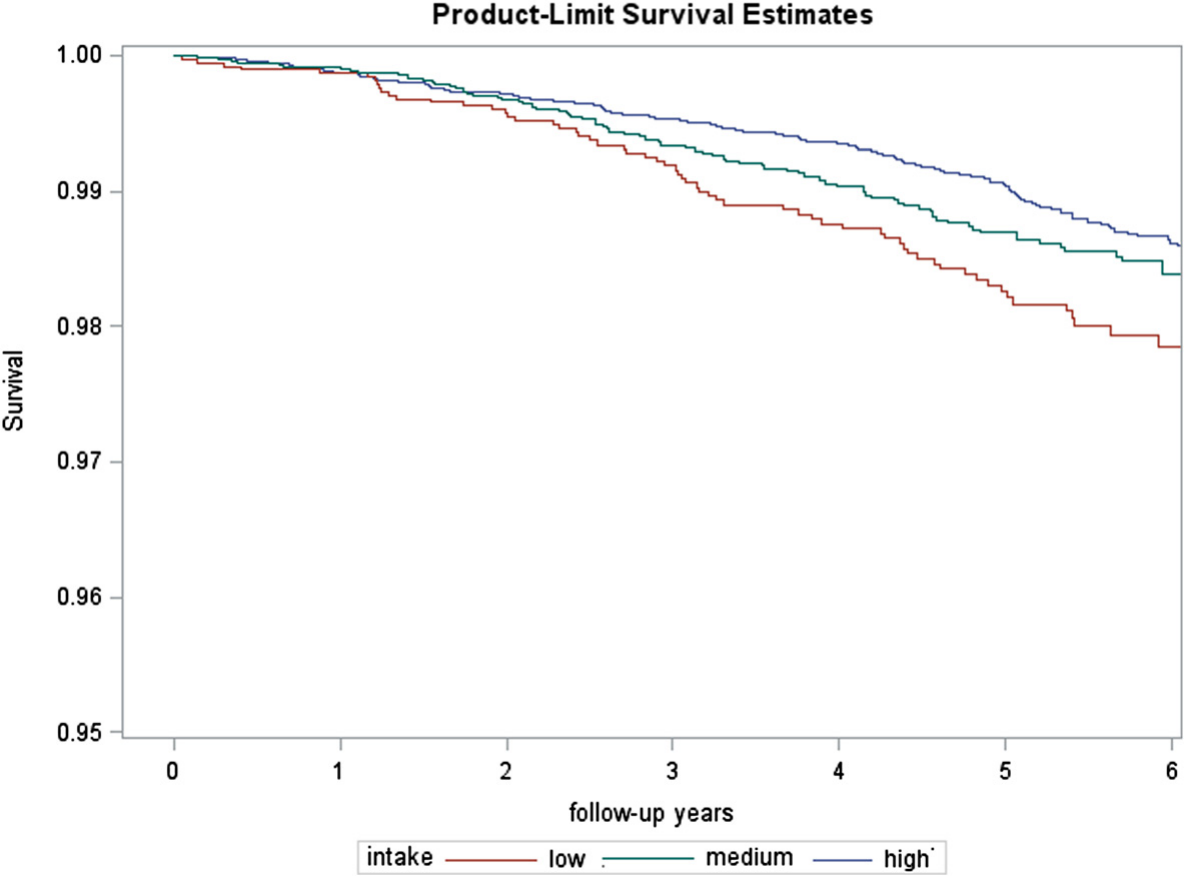
Nelson Aalen estimates of the incidence of death by groups of polyphenol intake.

During a mean of 4.8 years of follow-up among 31,068 person-years, the total number of observed deaths was 327. Of these, 131 were due to cancer, 81 were cardiovascular and 115 were for other causes. The Nelson Aalen survival function (Figure 1) shows the crude differences in death rates by groups of polyphenol intake: low (<600 mg/d), medium (600 to 750 mg/d) and high (>750 mg/d).

Table 2 shows Cox Proportional HRs and 95% CI for total mortality according to quintiles of cumulative intake of total polyphenols (according to yearly updated assessments). After adjusting for all potential confounders and stratifying by sex, recruitment center and intervention group, the HR for the highest versus the lowest quintile was 0.60 (95% CI, 0.39 to 0.91, *P*-trend = 0.07). After further adjustment for other dietary confounders, the association was not substantially attenuated (HR 0.63, 95% CI, 0.41 to 0.97, *P*-trend = 0.12). We did not see a strong inverse linear trend for total polyphenols; instead, the results suggest a modest threshold above the first quintile of intake.

**Table 2:** Cox proportional hazard ratios for total mortality according to quintiles of cumulative total polyphenol intake

	**Quintiles of cumulative intake of total polyphenols, mg/d**					
	**Q1 (535)**	**Q2 (700)**	**Q3 (800)**	**Q4 (917)**	**Q5 (1170)**	***P-*** **trend**
No. of deaths	88	62	52	63	62	
No. of person-years	5,505	6,599	6,767	6,559	5,638	
Age- and sex-adjusted HR (95% CI)^*^	1.00	0.65 (0.44 to 0.95)	0.55 (0.37 to 0.82)	0.73 (0.50 to 1.06)	0.66 (0.44 to 0.98)	0.12
Multivariable-adjusted HR (95% CI)^†^	1.00	0.68 (0.46 to 1.01)	0.60 (0.39 to 0.90)	0.75 (0.51 to 1.12)	0.60 (0.39 to 0.91)	0.07
Additionally adjusted HR (95% CI)^‡^	1.00	0.71 (0.48 to 1.05)	0.62 (0.41 to 0.95)	0.79 (0.53 to 1.17)	0.63 (0.41 to 0.97)	0.12

HR, Hazard ratio; CI, Confidence interval.

^*^Analyses were stratified by sex, recruitment center and intervention group.

^†^The multivariable HR has been additionally adjusted for age (<60, 60 to 64.9, 65 to 69.9, 70 to 74.9, >/=75 years), smoking (never, past and current: cigarettes (<5, 5 to 19, >20 per day) or cigars and pipes (<3, 3 to 6, >6 per day)), BMI (<25, 25 to 29.9, or >/=30 Kg/m^2^), baseline diabetes, alcohol (0, 0.1 to 14.9, 15 to 29.9, >/=30 g/day), total energy intake (continuous variable), physical activity (continuous variable), family history of CVD or cancer, aspirin use, antihypertensive drug use, use of cardiovascular medication, use of oral hypoglycaemic agents, insulin, other medication.

^‡^This model has been additionally adjusted for intake of protein, saturated fatty acids, polyunsaturated fatty acids, monounsaturated fatty acids and cholesterol (all as continuous variables).

In some cases, follow-ups were too short to assess a mortality endpoint because the ill-health conditions leading to death may influence diet. Therefore, as sensitivity analyses, we estimated the fully adjusted HR for the category of the highest total polyphenol intake vs. the lowest, excluding participants with less than one (31 excluded) or two years of follow-up (75 excluded). In both cases, the association was robust and remained statistically significant: HR 0.57, 95% CI, 0.36 to 0.90, *P*-trend = 0.07 and HR 0.49, 95% CI, 0.30 to 0.82, *P*-trend = 0.03, respectively.

We also conducted stratified analyses (Table 3) by the other strong predictors of mortality. In multivariable models, the inverse association between total polyphenol intake and risk of death, comparing the extreme quintiles, was stronger among women (HR 0.42, 95% CI, 0.18 to 0.98, *P*-trend = 0.24) than men (HR 0.76, 95% CI, 0.46 to 1.26, *P*-trend = 0.23), although the interaction for sex was not significant (*P*-interaction = 0.39). We also observed no significant differences by strata of age (<70 vs >/=70 years). However, we noted that those who did not drink alcohol had a stronger inverse association with total polyphenol intake (HR 0.39, 95% CI, 0.17 to 0.90, *P*-trend = 0.04) than drinkers (HR 0.99, 95% CI, 0.59 to 1.65, *P*-trend = 0.91), but the interaction was not significant (*P*-interaction = 0.16). In other stratified analyses, we observed that the inverse association did not change substantially among smokers and non-smokers, in those who were physically active or inactive, or in those with or without T2DM or hypertension, and none of these interactions were significant. Finally, we conducted stratified analyses by intervention groups and found a slightly stronger association between total polyphenol intake and death in the control arm of the trial (HR 0.48; CI 0.23 to 0.98; *P*-trend = 0.01) than in the MedDiet + EVOO arm (HR 0.67; CI 0.31 to 1.46; *P*-trend = 0.68) and the MedDiet + nuts arm (HR 0.68; CI 0.34 to 1.35; *P*-trend = 0.81). However, the interaction (*P* = 0.71) was not statistically significant, suggesting no apparent effect modification.

**Table 3:** HR for total mortality according to quintiles of total polyphenol intake (stratified by risk factors)

**Risk factor**	**No. of deaths**	**No. of person-years**	Multivariable-adjusted HR (95% CI), Quintile 5 vs. 1^*^	***P*-trend**	***P*-interaction**
**Sex**					
Men	203	13,317	0.76 (0.46 to 1.26)	0.23	0.39
Women	124	17,751	0.42 (0.18 to 0.98)	0.24	
**Age, y**					
<70	142	21,483	0.58 (0.31 to 1.08)	0.21	0.73
≥70	185	9,585	0.70 (0.39 to 1.24)	0.34	
**Alcohol intake**					
Nondrinkers	133	12,510	0.39 (0.17 to 0.90)	0.04	0.16
Drinkers	194	18,558	0.99 (0.59 to 1.65)	0.91	
**Smoking**					
Never	144	19,520	0.64 (0.31 to 1.32)	0.47	0.93
Former	111	7,465	0.52 (0.25 to 1.07)	0.29	
Current	72	4,083	0.71 (0.29 to 1.75)	0.21	
**Physical activity**					
Less than median	203	16,224	0.57 (0.32 to 1.02)	0.17	0.43
More than median	124	14,844	0.77 (0.41 to 1.44)	0.73	
**Hypertension**					
Yes	184	12,080	0.63 (0.36 to 1.10)	0.24	0.21
No	134	17,721	0.82 (0.44 to 1.55)	0.76	
**Diabetes mellitus**					
Yes	205	15,345	0.79 (0.47 to 1.33)	0.92	0.52
No	122	15,723	0.60 (0.31 to 1.17)	0.09	
**Intervention group**					
MedDiet-EVOO	113	11,478	0.67 (0.31 to 1.46)	0.68	0.71
MedDiet-Nuts	108	10,134	0.68 (0.34 to 1.35)	0.81	
Control Diet	106	9,456	0.48 (0.23 to 0.98)	0.01	

HR, Hazard ratio; CI, Confidence interval; MedDiet-EVOO, Mediterranean Diet supplemented with extra virgin olive oil; MedDiet-nuts, Mediterranean Diet supplemented with nuts.

^*^The multivariable HR has been additionally adjusted for age (<60, 60to 4.9, 65 to 69.9, 70 to 74.9, >/=75 years), smoking (never, past and current: cigarettes (<5, 5 to 19, >20 per day) or cigars and pipes (<3, 3 to 6, >6 per day)), BMI (<25, 25 to 29.9, or >/=30 Kg/m^2^), baseline diabetes, alcohol (0, 0.1 to 14.9, 15 to 29.9, >/=30 g/day), total energy intake (continuous variable), physical activity (continuous variable), family history of CVD or cancer, aspirin use, antihypertensive drug use, use of cardiovascular medication, use of oral hypoglycemic agents, insulin, other medication. Analyses were stratified by sex, recruitment center and intervention group.

We further investigated the possible effects of the intake of the main polyphenol groups on mortality by any cause (Table 4). Although no significant associations were found for flavonoids or phenolic acids, we observed a 46% reduction in risk of death in participants who consumed more stilbenes (HR 0.48; CI 0.25 to 0.91; *P*-trend = 0.04) and lignans (HR 0.60; CI 0.37 to 0.95; *P*-trend = 0.03). For “other polyphenols”, such as tyrosols, alkylphenols, hydroxybenzaldehydes, furanocoumarins and hydroxycoumarins, the association was attenuated after adjustment for other nutrients.

**Table 4:** Relationship between mortality and intake of the main polyphenol groups (in quintiles)

**Main groups**	**Q1**	**Q2**	**Q3**	**Q4**	**Q5**	***P*-trend**
**Flavonoids (mg/d)**	273	362	431	512	670	
No. of deaths	76	73	42	69	67	
No. of person-years	4,890	6,599	6,755	6,867	5,957	
Age- and sex-adjusted HR (95% CI)^*^	1.00	0.76 (0.52 to 1.10)^*^	0.54 (0.36 to 0.81)	0.72 (0.49 to 1.05)	0.70 (0.47 to 1.05)	0.23
Multivariable-adjusted HR (95% CI)^†^	1.00	0.92 (0.62 to 1.34)	0.69 (0.45 to 1.07)	0.92 (0.62 to 1.36)	0.83 (0.55 to 1.27)	0.70
Additionally adjusted HR (95% CI)^‡^	1.00	0.96 (0.65 to 1.41)	0.75 (0.48 to 1.16)	0.99 (0.66 to 1.47)	0.89 (0.58 to 1.36)	0.95
**Phenolic acids (mg/d)**	159	229	279	345	453	
No. of deaths	80	58	62	69	58	
No. of person-years	5,928	6,662	6,716	6,615	5,147	
Age- and sex-adjusted HR (95% CI)^*^	1.00	0.95 (0.65 to 1.39)	0.78 (0.53 to 1.16)	1.01 (0.70 to 1.47)	0.95 (0.63 to 1.42)	0.64
Multivariable-adjusted HR (95% CI)^†^	1.00	0.94 (0.64 to 1.39)	0.82 (0.55 to 1.23)	1.07 (0.72 to 1.58)	0.79 (0.51 to 1.22)	0.25
Additionally adjusted HR (95% CI)^‡^	1.00	0.89 (0.60 to 1.31)	0.77 (0.52 to 1.16)	1.01 (0.68 to 1.50)	0.75 (0.49 to 1.16)	0.20
**Stilbenes (mg/d)**	0	0.48	1.04	2.04	5.75	
No. of deaths	69	64	47	74	73	
No. of person-years	5,191	6,547	6,840	6,527	5,963	
Age- and sex-adjusted HR (95% CI)^*^	1.00	0.71 (0.47 to 1.05)	0.66 (0.44 to 0.98)	0.81 (0.56 to 1.18)	0.73 (0.56 to 1.18)	0.44
Multivariable-adjusted HR (95% CI)^†^	1.00	0.61 (0.33 to 1.11)	0.53 (0.28 to 0.99)	0.68 (0.38 to 1.22)	0.42 (0.22 to 0.81)	0.04
Additionally adjusted HR (95% CI)^‡^	1.00	0.69 (0.38 to 1.27)	0.62 (0.33 to 1.16)	0.78 (0.43 to 1.40)	0.48 (0.25 to 0.91)	0.04
**Lignans (mg/d)**	0.44	0.57	0.67	0.77	0.94	
No. of deaths	76	72	57	55	67	
No. of person-years	4,457	6,002	6,737	7,146	6,726	
Age- and sex-adjusted HR (95% CI)^*^	1.00	0.66 (0.46 to 0.96)	0.58 (0.39 to 0.85)	0.58 (0.39 to 0.87)	0.54 (0.35 to 0.82)	0.002
Multivariable-adjusted HR (95% CI)^†^	1.00	0.65 (0.44 to 0.99)	0.56 (0.38 to 0.84)	0.56 (0.36 to 0.84)	0.51 (0.32 to 0.79)	0.001
Additionally adjusted HR (95% CI)^‡^	1.00	0.68 (0.46 to 1.00)	0.60 (0.40 to 0.92)	0.62 (0.39 to 0.98)	0.60 (0.37 to 0.97)	0.03
**Others (mg/d)**	37	53	66	82	113	
No. of deaths	77	65	72	60	53	
No. of person-years	4,604	6,442	7,320	6,777	5,925	
Age- and sex-adjusted HR (95% CI)^*^	1.00	0.76 (0.52 to 1.11)	0.78 (0.54 to 1.13)	0.68 (0.46 to 1.01)	0.64 (0.42 to 0.96)	0.04
Multivariable-adjusted HR (95% CI)^†^	1.00	0.76 (0.51 to 1.13)	0.80 (0.54 to 1.18)	0.67 (0.45 to 1.02)	0.61 (0.40 to 0.93)	0.03
Additionally adjusted HR (95% CI)^‡^	1.00	0.82 (0.55 to 1.22)	0.86 (0.58 to 1.27)	0.76 (0.50 to 1.16)	0.70 (0.46 to 1.09)	0.13

HR*,* Hazard Ratio; CI*,* confidence interval.

^*^Analyses were stratified by sex, recruitment center and intervention group.

^†^The multivariable HR has been additionally adjusted for age (<60, 60 to 64.9, 65 to 69.9, 70 to 74.9, >/=75 years), smoking (never, past and current: cigarettes (<5, 5 to 19, >20 per day) or cigars and pipes (<3, 3 to 6, >6 per day)), BMI (<25, 25 to 29.9, or >/=30 Kg/m^2^), baseline diabetes, alcohol (0, 0.1 to 14.9, 15 to 29.9, >/=30 g/day), total energy intake (continuous variable), physical activity (continuous variable), family history of CVD or cancer, aspirin use, antihypertensive drug use, use of cardiovascular medication, use of oral hypoglycaemic agents, insulin, other medication.

^‡^This model has been additionally adjusted for intake of protein, saturated fatty acids, polyunsaturated fatty acids, monounsaturated fatty acids and cholesterol (all as continuous variables).

**Figure 2:**
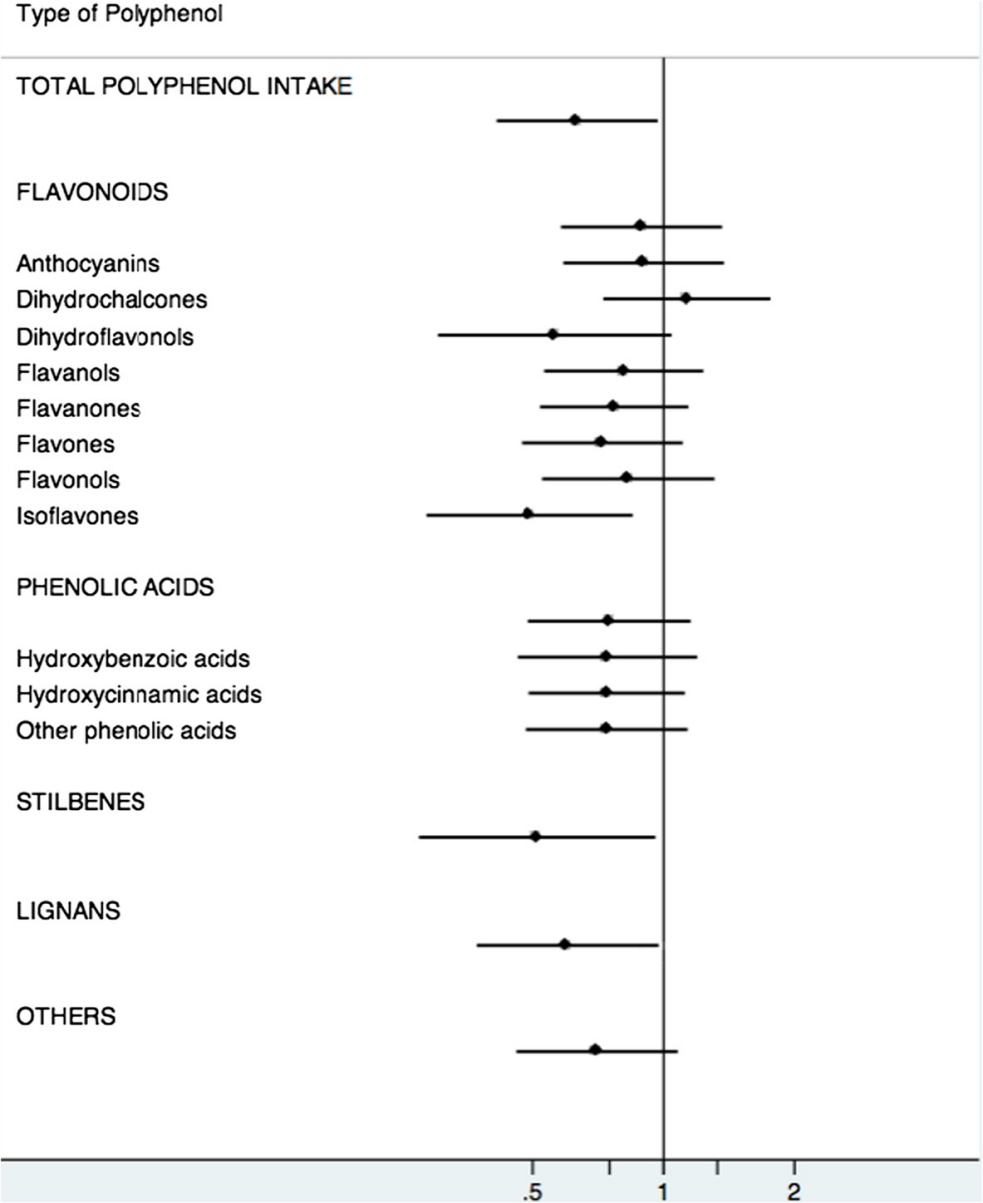
Hazard ratios (95% CI) of total mortality for the highest vs. lowest quintiles of polyphenol intake.

Exploratory analyses (Figure 2) were done for flavonoids (see Additional file 1) and phenolic acid subclasses (see Additional file 2). We found a strong trend towards a reduction in death risk with a higher intake of isoflavones (HR 0.49; CI 0.28 to 0.84; *P*-trend = 0.009). Dihydroflavonols were also inversely associated with the risk of death after multivariable adjustment (HR 0.53; CI 0.28 to 0.99; *P*-trend = 0.05) and the inverse trend was statistically significant after additional adjustment (*P*-trend = 0.04). No other subclasses were associated with mortality by any cause.

## Discussion

In this reanalysis of the data of the PREDIMED trial, we observed a 37% reduction of mortality when comparing extreme quintiles of total polyphenol intake. The dose-response trend for the association between total polyphenol intake and all-cause mortality suggested an L-shaped relationship, with an apparent threshold after the first quintile of polyphenol intake, instead of an inverse linear dose-response relationship. Within the polyphenol subclasses, stilbenes and lignans were inversely associated with total mortality.

In stratified analyses we found a stronger association between total polyphenol intake and mortality risk for women and for those who did not drink alcohol. Although the interaction terms were not significant, the observed trend was suggestive, especially for non-drinkers. The relationship between alcohol intake and polyphenols should be the main focus of future studies.

To our knowledge, though previous studies have investigated the association between intake of specific groups of polyphenols and mortality, this is the first study to investigate the association between total polyphenol intake, as well as that of all polyphenol subgroups with all-cause mortality. In addition, we should acknowledge that the effect of polyphenols and polyphenol-rich foods on chronic degenerative diseases and clinical biomarkers has been broadly studied [19-24]. Previous studies have analyzed the association between polyphenols from wine, tea, chocolate, berries, soy and olive oil with several chronic degenerative disease risk or mortality risk [6,25-29]. The reported inverse association, specifically for olive oil and red wine, is consistent with the inverse association we found for stilbenes and lignans [29-31]. The suggestion of an inverse association that we found for several flavonoid compounds is also consistent with previous studies of berries, dark chocolate and soy [6,25,26]. In many of these previously studied populations, intake of any one polyphenol-rich food was not great enough to reduce mortality, but in our study total polyphenol intake was a wider range, coming from several food sources.

Kuriyama *et al*. conducted a prospective cohort study among 40,530 healthy Japanese adults and reported that green tea consumption, a polyphenol-rich beverage, was inversely associated with cardiovascular diseases and all-cause mortality, but not with mortality due to cancer [27]. Other studies have also found an inverse association between polyphenol consumption and CVD and CVD-related mortality [20,25,26,32]. Indeed, it has been demonstrated that some polyphenols and their metabolites exert anti-atherosclerotic effects, improve endothelial function and antioxidant status, increase nitric oxide release, and modulate inflammation and lipid metabolism [5,21,25,33-35].

Polyphenols can also act as chemopreventive agents. For example, resveratrol is a well-known stilbene, mostly found in red wine and grapes, with several health benefits, including inhibition of tumorgenesis [8,36,37]. *In vitro* and *in vivo* studies have shown that epigallocatechin-3-gallate, the major polyphenol of green tea, has anti-carcinogenic effects, such as inhibition of growth proliferation, induction of apoptosis and phase II detoxifying enzymes, and reduction of oxidative damage to DNA [36-38]. Xanthohumol, quercetin, curcumin and genistein are other examples of polyphenols that have shown anticarcinogenic properties due to their capacity to inhibit tumor growth [8,22,37,38].

Available evidence supports that dietary modifications are able to reduce the risk of T2DM, another highly prevalent chronic disease. Wedick *et al*. found that anthocyanins were inversely associated with the risk of T2DM using data from three US prospective cohorts and Muraki *et al*. found similar associations for blueberries, grapes and apples [39,40]. Finally, polyphenols have been proposed as promising phytochemicals for the treatment and prevention of neurogenerative diseases such as Alzheimer’s disease, Parkinson’s disease and other neurological disorders [29,41].

All of this evidence from chronic disease studies supports the hypothesis that greater polyphenol intake, and the many polyphenol subclasses this represents, serves to extend the life span through multifactorial etiological pathways.

Our study has some limitations. First, we controlled for several confounders in multivariate models, but other unknown or unmeasured confounders may exist. However, if this were the case, we would expect relative risks for all subclasses to be equally over or underestimated and that was not the case. Second, the number of cases of cause-specific deaths was too low to estimate individual relative risks. Others have found the benefits of specific foods are stronger for CVD mortality than cancer or respiratory disease. Future work in this area should include larger studies with estimates of total polyphenol intake. Third, there were limitations with respect to the estimation of polyphenol intake because data were indirectly derived from the FFQs. Although urinary excretion of polyphenols was validated as a biomarker of total polyphenol from the FFQ in two different studies, the values of r were relatively low. The absence of information about some foods in the FFQ could lead to an underestimation of the intake. Moreover, the study did not take into account the bioavailability of these molecules. Finally, these results might be valid only for elderly people at high cardiovascular risk and other studies are needed to generalize the conclusions to other populations.

On the other hand, the main strengths of the study are the prospective design, the large sample size with a relatively long-term follow-up, and comprehensive data on risk factors and confounders. Very importantly, our use of the cumulative average of polyphenol intake across yearly repeated measurements of diet is considered as the best approach to reduce measurement error in nutritional epidemiology [42] and allowed changes in the diet due to the intervention or other secular trends in intake in Spain to be controlled. We also used the most comprehensive polyphenol database currently available (Phenol-explorer database), which allowed risk estimation related not only to intake of total polyphenol but also all the polyphenol subgroups and subclasses. This comprehensive analysis differentiates our paper from previous related studies.

## Conclusions

We found an apparent inverse association between total polyphenol intake and the risk of overall mortality, which was independent of other dietary and non-dietary risk factors. This may be helpful in establishing future daily polyphenol intake recommendations. However, more studies are needed to definitively clarify the benefits deriving from long-term consumption of polyphenol-rich foods.

### Other PREDIMED Investigators

Other contributors list (Additional file 3).

## Abbreviations

ANOVA: Analysis of Variance; BMI: Body Mass Index; CVD: Cardiovascular diseases; EVOO: Extra Virgin Olive Oil; FFQ: Food Frequency Questionnaire; HR: Hazard ratio; MedDiet: Mediterranean Diet; PREDIMED: Prevención con Dieta Mediterránea; SD: Standard deviation; T2DM: Type 2 diabetes mellitus; 95% CI: 95% Confidence interval.

## Competing interests

The authors declare that they have no competing interests.

## Authors’ contributions

ATR, RMLR, EBR, RE and MAMG carried out the statistical analyses, interpreted the data and drafted the manuscript. RMLR, RE, MAMG, AMR, MCLS, MIC, DC, JSS, EGG, JL, FA, MF, ER, LSM, XP, MAM, AG and VRG participated in the design of the study and the acquisition of data and contributed to the critical review of the paper. All authors read and approved the final manuscript.

## Supplementary Material

Additional file 1 Flavonoids.doc. Click here for file

Additional file 2 Phenolic acids.doc. Click here for file

Additional file 3 Other contributors’ list.doc. Click here for file
