# Supplementary material for: Polyphenol intake and mortality risk: a re-analysis of the PREDIMED trial
Source: BMC Med. 2014 May 13;12:77. doi: 10.1186/1741-7015-12-77 (PMC4102266; doi:10.1186/1741-7015-12-77)
Supplement: Additional file 1 — Flavonoids.doc. [file 1741-7015-12-77-S1.docx]

**Additional file 1.**The relationship between mortality and flavonoid subclass intake (in quintiles).

| **Flavonoids** | Q1 | Q2 | Q3 | Q4 | Q5 | *P* value for Trend |
| --- | --- | --- | --- | --- | --- | --- |
| **Anthocyanins (mg/d)** | 11.8 | 23.6 | 32.8 | 45.7 | 74.6 |  |
| No. of deaths | 81 | 63 | 53 | 50 | 80 |  |
| No. of person-years | 5886 | 6488 | 6503 | 6409 | 5782 |  |
| Age- and sex-adjusted HR (95% CI) ^*^ | 1.00 | 0.76 (0.52-1.10) ^*^ | 0.68 (0.46-1.01) | 0.59 (0.39-0.88) | 0.95 (0.66-1.37) | 0.95 |
| Multivariable-adjusted HR (95% CI) ^†^ | 1.00 | 0.68 (0.46-1.01) | 0.71 (0.47-1.08) | 0.57 (0.37-0.87) | 0.89 (0.58-1.35) | 0.79 |
| Additionally adjusted HR (95% CI) ^‡^ | 1.00 | 0.68 (0.46-1.01) | 0.73 (0.48-1.12) | 0.56 (0.36-0.87) | 0.90 (0.59-1.38) | 0.84 |
| **Dihydrochalcones (mg/d)** | 0.8 | 1.8 | 2.6 | 3.5 | 5.8 |  |
| No. of deaths | 67 | 59 | 68 | 65 | 68 |  |
| No. of person-years | 5302 | 6329 | 7112 | 5653 | 6673 |  |
| Age- and sex-adjusted HR (95% CI) ^*^ | 1.00 | 0.99 (0.66-1.48) | 0.86 (0.57-1.28) | 0.99 (0.66-1.49) | 0.92 (0.61-1.40) | 0.77 |
| Multivariable-adjusted HR (95% CI) ^†^ | 1.00 | 1.07 (0.70-1.63) | 0.98 (0.66-1.49) | 1.04 (0.68-1.60) | 1.07 (0.69-1.65) | 0.81 |
| Additionally adjusted HR (95% CI) ^‡^ | 1.00 | 1.07 (0.70-1.64) | 1.02 (0.67-1.57) | 1.13 (0.73-1.74) | 1.13 (0.73-1.77) | 0.58 |
| **Dihydroflavonols (mg/d)** | 0.1 | 1.4 | 2.3 | 3.8 | 9.8 |  |
| No. of deaths | 68 | 65 | 48 | 73 | 73 |  |
| No. of person-years | 5130 | 6577 | 6880 | 6528 | 5954 |  |
| Age- and sex-adjusted HR (95% CI) ^*^ | 1.00 | 0.91 (0.61-1.34) | 0.66 (0.44-1.00) | 0.88 (0.60-1.29) | 0.79 (0.53-1.18) | 0.44 |
| Multivariable-adjusted HR (95% CI) ^†^ | 1.00 | 0.90 (0.52-1.54) | 0.61 (0.33-1.14) | 0.86 (0.49-1.53) | 0.53 (0.28-0.99) | 0.05 |
| Additionally adjusted HR (95% CI) ^‡^ | 1.00 | 0.97 (0.57-1.66) | 0.67 (0.36-1.24) | 0.92 (0.52-1.62) | 0.56 (0.30-1.04) | 0.04 |
| **Flavanols (mg/d)** | 90 | 129 | 158 | 192 | 263 |  |
| No. of deaths | 89 | 50 | 62 | 59 | 67 |  |
| No. of person-years | 5174 | 6280 | 6754 | 6780 | 6080 |  |
| Age- and sex-adjusted HR (95% CI) ^*^ | 1.00 | 0.50 (0.34-0.75) | 0.62 (0.43-0.89) | 0.60 (0.42-0.87) | 0.62 (0.42-0.91) | 0.06 |
| Multivariable-adjusted HR (95% CI) ^†^ | 1.00 | 0.55 (0.36-0.83) | 0.71 (0.48-1.05) | 0.67 (0.45-0.99) | 0.73 (0.48-1.12) | 0.32 |
| Additionally adjusted HR (95% CI) ^‡^ | 1.00 | 0.60 (0.39-0.91) | 0.77 (0.52-1.14) | 0.75 (0.50-1.12) | 0.81 (0.53-1.23) | 0.60 |
| **Flavanones (mg/d)** | 28 | 78 | 113 | 157 | 247 |  |
| No. of deaths | 84 | 61 | 62 | 54 | 66 |  |
| No. of person-years | 4659 | 5663 | 7386 | 6466 | 6894 |  |
| Age- and sex-adjusted HR (95% CI) ^*^ | 1.00 | 0.64 (0.43-0.93) | 0.54 (0.37-0.78) | 0.51 (0.34-0.77) | 0.61 (0.42-0.89) | 0.02 |
| Multivariable-adjusted HR (95% CI) ^†^ | 1.00 | 0.71 (0.48-1.05) | 0.68 (0.46-0.99) | 0.65 (0.45-0.98) | 0.73 (0.50-1.07) | 0.15 |
| Additionally adjusted HR (95% CI) ^‡^ | 1.00 | 0.75 (0.51-1.17) | 0.70 (0.48-1.04) | 0.69 (0.46-1.05) | 0.77 (0.52-1.14) | 0.25 |
| **Flavones (mg/d)** | 20 | 29 | 37 | 46 | 67 |  |
| No. of deaths | 78 | 69 | 62 | 60 | 58 |  |
| No. of person-years | 4822 | 6213 | 6592 | 7077 | 6364 |  |
| Age- and sex-adjusted HR (95% CI) ^*^ | 1.00 | 0.75 (0.52-1.09) | 0.76 (0.52-1.11) | 0.71 (0.48-1.04) | 0.63 (0.42-0.95) | 0.04 |
| Multivariable-adjusted HR (95% CI) ^†^ | 1.00 | 0.82 (0.56-1.21) | 0.93 (0.63-1.38) | 0.85 (0.57-1.27) | 0.71 (0.46-1.07) | 0.14 |
| Additionally adjusted HR (95% CI) ^‡^ | 1.00 | 0.83 (0.56-1.22) | 0.96 (0.64-1.43) | 0.87 (0.58-1.31) | 0.72 (0.47-1.11) | 0.18 |
| **Flavonols (mg/d)** | 56 | 74 | 88 | 101 | 124 |  |
| No. of deaths | 84 | 73 | 67 | 50 | 53 |  |
| No. of person-years | 6053 | 6909 | 6360 | 6214 | 5532 |  |
| Age- and sex-adjusted HR (95% CI) ^*^ | 1.00 | 0.84 (0.58-1.20) | 0.80 (0.55-1.17) | 0.56 (0.37-0.87) | 0.61 (0.40-0.95) | 0.01 |
| Multivariable-adjusted HR (95% CI) ^†^ | 1.00 | 1.00 (0.69-1.44) | 0.89 (0.61-1.31) | 0.65 (0.41-1.01) | 0.70 (0.45-1.10) | 0.06 |
| Additionally adjusted HR (95% CI) ^‡^ | 1.00 | 1.06 (0.73-1.54) | 0.96 (0.65-1.42) | 0.72 (0.46-1.14) | 0.83 (0.53-1.32) | 0.26 |
| **Isoflavones (mg/d)** | 0.011 | 0.018 | 0.024 | 0.034 | 0.050 |  |
| No. of deaths | 75 | 74 | 72 | 59 | 47 |  |
| No. of person-years | 4958 | 6073 | 6648 | 6831 | 6559 |  |
| Age- and sex-adjusted HR (95% CI) ^*^ | 1.00 | 0.95 (0.66-1.38) | 0.82 (0.57-1.19) | 0.49 (0.32-0.75) | 0.26 (0.15-0.43) | <0.001 |
| Multivariable-adjusted HR (95% CI) ^†^ | 1.00 | 1.01 (0.68-1.51) | 0.92 (0.61-1.38) | 0.67 (0.43-1.04) | 0.35 (0.21-0.60) | <0.001 |
| Additionally adjusted HR (95% CI) ^‡^ | 1.00 | 1.10 (0.73-1.64) | 1.03 (0.68-1.55) | 0.80 (0.51-1.25) | 0.49 (0.28-0.84) | 0.009 |

Abbreviation: HR, Hazard Ratio; CI, confidence interval

^*^ Analyses were stratified by sex, recruitment centre and intervention group.

^†^ The multivariable HR has been additionally adjusted for age (<60, 60-64.9, 65-69.9, 70-74.9, >=75 years), smoking (never, past and current: cigarettes (<5, 5-19, >20 per day) or cigars and pipes (<3, 3-6, >6 per day)), BMI (<25, 25-29.9, or >=30 Kg/m^2^), baseline diabetes, alcohol (0, 0.1-14.9, 15-29.9, >=30 g/day), total energy intake (continuous variable), physical activity (continuous variable), family history of CVD or cancer, aspirin use, antihypertensive drug use, use of cardiovascular medication, use of oral hypoglycaemic agents, insulin, other medication.

^‡^ This model has been additionally adjusted for intake of protein, saturated fatty acids, polyunsaturated fatty acids, monounsaturated fatty acids, and cholesterol (all as continuous variables).
