# Supplementary material for: Polyphenol intake and mortality risk: a re-analysis of the PREDIMED trial
Source: BMC Med. 2014 May 13;12:77. doi: 10.1186/1741-7015-12-77 (PMC4102266; doi:10.1186/1741-7015-12-77)
Supplement: Additional file 2 — Phenolic acids.doc. [file 1741-7015-12-77-S2.docx]

**Additional file 2able 54th to Nov, 29 ...rspi + writing classcardiovascular en la cohorte del PREDIMED.oviembre..** The relationship between mortality and phenolic acid subclasses intake (in quintiles).

| Phenolic acids | Q1 | Q2 | Q3 | Q4 | Q5 | *P* value for Trend |
| --- | --- | --- | --- | --- | --- | --- |
| **Hydroxybenzoic acids (mg/d)** | 6.9 | 12.9 | 17.8 | 24.1 | 36.1 |  |
| No. of deaths | 80 | 74 | 50 | 56 | 67 |  |
| No. of person-years | 5539 | 6727 | 6734 | 6738 | 5330 |  |
| Age- and sex-adjusted HR (95% CI) ^*^ | 1.00 | 0.89 (0.62-1.29) ^*^ | 0.62 (0.41-0.92) | 0.59 (0.39-0.88) | 0.70 (0.47-1.04) | 0.04 |
| Multivariable-adjusted HR (95% CI) ^†^ | 1.00 | 0.83 (0.56-1.22) | 0.60 (0.39-0.91) | 0.54 (0.35-0.84) | 0.58 (0.37-0.93) | 0.01 |
| Additionally adjusted HR (95% CI) ^‡^ | 1.00 | 0.90 (0.61-1.34) | 0.68 (0.44-1.04) | 0.66 (0.42-1.04) | 0.74 (0.46-1.20) | 0.17 |
| **Hydroxycinnamic acids (mg/d)** | 138 | 207 | 252 | 316 | 422 |  |
| No. of deaths | 81 | 58 | 57 | 66 | 65 |  |
| No. of person-years | 5941 | 6621 | 6543 | 6776 | 5186 |  |
| Age- and sex-adjusted HR (95% CI) ^*^ | 1.00 | 0.71 (0.48-1.04) | 0.78 (0.53-1.14) | 0.80 (0.55-1.16) | 0.97 (0.66-1.43) | 0.91 |
| Multivariable-adjusted HR (95% CI) ^†^ | 1.00 | 0.67 (0.45-0.99) | 0.75 (0.51-1.11) | 0.75 (0.51-1.11) | 0.78 (0.52-1.18) | 0.42 |
| Additionally adjusted HR (95% CI) ^‡^ | 1.00 | 0.63 (0.42-0.93) | 0.71 (0.48-1.06) | 0.71 (0.48-1.05) | 0.74 (0.49-1.12) | 0.32 |
| **Other phenolic acids (mg/d)** | 0.1 | 2.5 | 4.6 | 8.6 | 17.9 |  |
| No. of deaths | 77 | 62 | 58 | 67 | 63 |  |
| No. of person-years | 5199 | 6485 | 6608 | 7555 | 5221 |  |
| Age- and sex-adjusted HR (95% CI) ^*^ | 1.00 | 0.76 (0.52-1.12) | 0.68 (0.45-1.01) | 0.76 (0.52-1.12) | 0.83 (0.56-1.24) | 0.77 |
| Multivariable-adjusted HR (95% CI) ^†^ | 1.00 | 0.64 (0.42-0.97) | 0.57 (0.37-0.89) | 0.68 (0.45-1.02) | 0.68 (0.44-1.03) | 0.38 |
| Additionally adjusted HR (95% CI) ^‡^ | 1.00 | 0.67 (0.44-1.03) | 0.61 (0.39-0.95) | 0.72 (0.48-1.09) | 0.74 (0.48-1.13) | 0.60 |

Abbreviation: HR, Hazard Ratio; CI, confidence interval

^*^ Analyses were stratified by sex, recruitment centre and intervention group.

^†^ The multivariate HR has been additionally adjusted for age (<60, 60-64.9, 65-69.9, 70-74.9, >=75 years), smoking (never, past and current: cigarettes (<5, 5-19, >20 per day) or cigars and pipes (<3, 3-6, >6 per day)), BMI (<25, 25-29.9, or >=30 Kg/m^2^), baseline diabetes, alcohol (0, 0.1-14.9, 15-29.9, >=30 g/day), total energy intake (continuous variable), physical activity (continuous variable), family history of CVD or cancer, aspirin use, antihypertensive drug use, use of cardiovascular medication, use of oral hypoglycaemic agents, insulin, other medication.

^‡^ This model has been additionally adjusted for intake of protein, saturated fatty acids, polyunsaturated fatty acids, monounsaturated fatty acids, and cholesterol (all as continuous variables).
