# Supplementary material for: Polyphenol intake and mortality risk: a re-analysis of the PREDIMED trial
Source: BMC Med. 2014 May 13;12:77. doi: 10.1186/1741-7015-12-77 (PMC4102266; doi:10.1186/1741-7015-12-77)
Supplement: Additional file 3 — Other contributors’ list.doc. [file 1741-7015-12-77-S3.docx]

**Hospital Clinic, Institut d’Investigacions Biomèdiques August Pi i Sunyer, Barcelona, Spain:** M. Serra, A. Pérez-Heras, C. Viñas, R. Casas, L. de Santamaría, S. Romero, E. Sacanella, G. Chiva. P. Valderas, S. Arranz, J.M. Baena, M. García, M. Oller, J. Amat, I. Duaso, Y. García, C. Iglesias, C. Simón, Ll. Quinzavos, Ll. Parra, M. Liroz, J. Benavent,  J. Clos, I. Pla, M. Amorós, M.T. Bonet, M.T. Martin, M.S. Sánchez, J. Altirriba, E. Manzano, A. Altés, M. Cofán, C. Valls-Pedret, A. Sala-Vila, M. Doménech.



**University of Navarra, Primary Care Centres, Pamplona, Spain:** A. Sánchez-Tainta, B. Sanjulián, E. Toledo, M. Bes-Rastrollo, A. Martí, P. Buil-Cosiales, M. Serrano-Martínez, J. Díez-Espino, A. García-Arellano, I. Zazpe, F.J. Basterra-Gortari, A. Gea, M. Garcia-Lopez, J.M. Nuñez-Córdoba, E. Cabello-Saavedra, N. Berrade, V.
Extremera-Urabayen, C. Arroyo-Azpa, L García-Pérez, A. Hernandez-Hernandez, L. Fanlo-Blasco, M.T. Lacalle Larrea, A. Lezaun-Indurain, A. Arillo-Crespo, E. Arina Vergara, M.L. Barandiaran Bengoetxea.

**University Rovira i Virgili, Reus, Spain:** M. Bulló, R. González, C. Molina, F. Márquez, N. Babio, M. Sorli, J. García-Roselló, F. Martin, R. Tort, A. Isach, R.Sagarra, J.J. Cabré, J. Fernández-Ballart, N. Ibarrola, M. Juanola-Falgarona, A. Díaz-López, M. Guasch-Ferré, C. Alegret, P. Martínez, S. Millán, J.L.Piñol, and J.M. Hernández, P. Castro, JM. Roca, M. Perez-Bauer, M. Vaquer, D. Gil, Aragonés E.

**Institut de Recerca Hospital del Mar, Barcelona, Spain:** M. Covas, S. Tello,
J. Vila, M. Fitó, H. Schröder, , D. Muñoz-Aguayo, R. Elosúa, J. Marrugat,
and M. Ferrer.

**University of Valencia, Valencia, Spain:** P. Carrasco, R. Osma, M. Guillén,
P. Guillem-Saiz, O. Portolés, V. Pascual, C. Riera, J. Valderrama, A.
Serrano, E. Lázaro, A. Sanmartín, A. Girbés, V. Santamaría, C. Sánchez, Z.
Plá, E.  Sánchez, C. Ortega-Azorín, J.I. González,  C. Saiz, O.Coltell and
E.M. Asensio.

**University Hospital of Alava, Vitoria, Spain:** I. Salaverría, J. San Vicente, A. Alonso-Gómez, A. Loma-Osorio, J. Rekondo, MC. Belló, J. Urraca

**University of Málaga, Málaga, Spain:** R. Benítez Pont, M. Bianchi Alba, J.
Fernández-Crehuet Navajas, J. Wärnberg, R. Gómez-Huelgas, J.
Martínez-González, V. Velasco García, J. de Diego Salas, A. Baca Osorio, J.
Gil Zarzosa, J.J. Sánchez Luque, and E. Vargas López.

**Instituto de la Grasa, Consejo Superior de Investigaciones Científicas,
Sevilla, Spain:** J. Sánchez Perona, E. Montero Romero, M. García García, and
E. Jurado Ruiz.

**Institute of Health Sciences IUNICS, University of Balearic Islands, and
Hospital Son Espases, Palma de Mallorca, Spain:** M. García-Valdueza, M.
Moñino, A. Proenza, R. Prieto, G. Frontera, M. Ginard, F. Fiol, A. Jover,
and J. García.

**Department of Family Medicine, Primary Care Division of Sevilla, Sevilla,
Spain:** M. Leal, E. Martínez, J.M. Santos, M. Ortega-Calvo, P. Román, F. José
García, P. Iglesias, Y. Corchado, L. Mellado, and N. Romero.

**School of Pharmacy, University of Barcelona, Barcelona, Spain:** M.C.
López-Sabater, A.I. Castellote-Bargallo.

**University of Las Palmas de Gran Canaria, Las Palmas, Spain:** J.Álvarez-Pérez, E. Díez-Benítez, A.Sánchez-Villegas, I. Bautista-Castaño, I. Maldonado-Díaz, P. Henriquez, C. Ruano, F. Sarmiendo-de la Fe, B. Macías-Gutiérrez, and A.J. Santana-Santana.

**University Hospital of Bellvitge, Hospitalet de Llobregat, Barcelona,
Spain:** E. de la Cruz, A. Galera, Y. Soler, F. Trias, I. Sarasa, E. Padres,
R. Figueras, X. Solanich, R. Pujol and E. Corbella.

**Primary Care Division, Catalan Institute of Health, Barcelona, Spain:** M.A. Muñoz-Pérez, C. Cabezas, M.A. Rovira, L. García, G. Flores, J.M. Verdú, P. Baby, A. Ramos, L. Mengual, P. Roura, M.C. Yuste, A. Guarner, A. Rovira, M.I. Santamaría, M. Mata, C. de Juan, A. Brau, and N. Molina.

**Department of Biochemistry and Molecular Biology, School of Medicine, University of Valencia, Valencia, Spain:** G.T. Sáez, C. Cerdá, A. Iradi, and C.Tormos.

**Other investigators of the PREDIMED network:** M.T. Mitjavila (University of
Barcelona), M.P. Portillo (University of Basque Country)
